# SR9009 improves heart function after pressure overload independent of cardiac REV-ERB

**DOI:** 10.3389/fcvm.2022.952114

**Published:** 2022-07-14

**Authors:** Hui Li, Shiyang Song, Chih-liang Tien, Lei Qi, Andrea Graves, Eleni Nasiotis, Thomas P. Burris, Yuanbiao Zhao, Zheng Sun, Lilei Zhang

**Affiliations:** ^1^Department of Molecular and Human Genetics, Baylor College of Medicine, Houston, TX, United States; ^2^Division of Diabetes, Department of Medicine, Endocrinology and Metabolism, Baylor College of Medicine, Houston, TX, United States; ^3^Genetics Institute, University of Florida, Gainesville, FL, United States; ^4^Department of Molecular and Cellular Biology, Baylor College of Medicine, Houston, TX, United States

**Keywords:** circadian clock, REV-ERB, SR9009, TAC, heart disease

## Abstract

The core clock component REV-ERB is essential for heart function. Previous studies show that REV-ERB agonist SR9009 ameliorates heart remodeling in the pressure overload model with transverse aortic constriction (TAC). However, it is unknown whether SR9009 indeed works through cardiac REV-ERB, given that SR9009 might target other proteins and that REV-ERB in non-cardiac tissues might regulate cardiac functions indirectly. To address this question, we generated the REV-ERBα/β cardiac-specific double knockout mice (cDKO). We found that REV-ERB cardiac deficiency leads to profound dilated cardiac myopathy after TAC compared to wild-type (WT) control mice, confirming the critical role of REV-ERB in protecting against pressure overload. Interestingly, the cardioprotective effect of SR9009 against TAC retains in cDKO mice. In addition, SR9009 administered at the time points corresponding to the peak or trough of REV-ERB expression showed similar cardioprotective effects, suggesting the REV-ERB-independent mechanisms in SR9009-mediated post-TAC cardioprotection. These findings highlight that genetic deletion of REV-ERB in cardiomyocytes accelerates adverse cardiac remodeling in response to pressure overload and demonstrated the REV-ERB-independent cardioprotective effect of SR9009 upon pressure overload.

## Introduction

Most living organisms’ behavior and physiological processes oscillate in day/night cycles. Disruption of the circadian rhythm has been well associated with cardiovascular disease, as exemplified by studies of shift workers (1, 2). In mammalian systems, the central clock exists in the suprachiasmatic nucleus (SCN) of the brain, while peripheral clocks outside the SCN exist and function in almost all cell types throughout the body. The function of the peripheral clocks, including those in the heart, has been increasingly appreciated from murine studies using tissue-specific peripheral clock deletion models (3–5).

The molecular clock is comprised of a transcriptional-translational feedback loop with the conserved core clock factors, including the transcription activators BMAL1/CLOCK and transcription repressor CRY/PER. REV-ERBα/REV-ERBβ are nuclear receptors with heme as the physiological ligand (6, 7), which stabilize and enhance the core clock. They are thought to act primarily as transcriptional repressors due to their lack of an activation domain, although recent work has shown that they may “tether” with other transcription factors for target recognition (8, 9).

The function of REV-ERB in the heart was initially established by a series of works using a pharmacological tool drug, SR9009. REV-ERB agonist was shown to protect cardiac function after pressure overload and myocardial infarction (10–12). Recently, we and others have demonstrated the physiological function of cardiac REV-ERB by constructing REV-ERBα/β double cardiac knockout mice (cDKO) that present progressive dilated cardiomyopathy (13, 14). In addition, we have shown that an abnormal temporal pattern of clock gene expression correlates with the severity of cardiac dilation in patients with idiopathic dilated cardiomyopathy (14).

It remains undetermined to what degree the cardioprotective effect of SR9009 is dependent on cardiac REV-ERB, considering that SR9009, like many small molecules, has off-target effects (15). The relative functional importance of REV-ERBα vs. REV-ERBβ in the heart is also unclear. REV-ERBα and REV-ERBβ are encoded by two different genes with a high homology (16, 17). Previous literature suggests that in most systems *Nr1d1* is dominant with some overlapping functions between the two (17–19).

Here we show that mice with REV-ERBα/β cardiac-specific double deletion (cDKO) are exquisitely sensitive to pressure overload and display a rapid onset of lethal dilated cardiomyopathy upon TAC as compared to the wild-type (WT) control. In comparison, REV-ERBβ single KO mice show a very mild phenotype, suggesting that REV-ERBα is dominant or there is significant functional redundancy between REV-ERBα and REV-ERBβ. We have found that SR9009 remains cardioprotective in cDKO mice compared to WT mice, indicating that cardiomyocyte REV-ERB is not required for the cardioprotection effect of SR9009. We also show that anti-phasic administration of SR9009 has similar effects to phasic administration, suggesting its effect is unlikely through REV-ERB in other cell types in the heart.

## Materials and methods

### Animals

Wild-type C57BL/6J mice were purchased from the Jackson Laboratory at the age of 7 weeks and allowed to acclimate in the Baylor College of Medicine for 2 weeks prior to the experiments described below. REV-ERBα and β floxed mice were previously described (Rev-erbα^loxP^ (*Nr1d1*^TM 1.2*Rev*^, MGI ID 5426700) and Rev-erbβ^loxP^ (*Nr1d2*^TM 1.1Rev^, MGI ID 5426699) (14). They were crossed to generate the double floxed mouse line (*Nr1d1/2^fl/fl^*). Exons 3 and 4 of *Nr1d1* were floxed, which leads to an in-frame deletion of the DNA binding domain upon Cre recombinase cleavage (20). Exon 4 of *Nr1d2* was floxed,^1^ which led to a frameshift deletion and nonsense-mediated decay of the transcript upon Cre recombinase cleavage (20). All the animal procedures were approved by the Institutional Animal Care and Use Committee at Baylor College of Medicine.

### Preparation and administration of SR9009

SR9009 was synthesized and purified in the laboratory of Thomas Burris (Department of Pharmacology and Physiology, St. Louis University, St. Louis, MO, United States) as previously published (13). For *in vivo* experiments, SR9009 was dissolved in 5% DMSO/10% Cremophor EL (Sigma-Aldrich, C5135)/85% PBS in a working solution at 10 mg/ml. Mice were injected at a dose of 100 mg/kg/day given i.p. once daily at zeitgeber time 6 or 18 (ZT6 and ZT18) as indicated. The diluent without SR9009 of the same volume was used as the control.

### Pressure overload (TAC)

All mice were C57BL/6J littermate males aged 9 weeks at the start of the experiment. Mice were anesthetized with 1% inhalational isoflurane, mechanically ventilated (Harvard apparatus), and subjected to thoracotomy. The aortic arch was constricted between the left and right carotid arteries using a 7.0 silk suture and a 27 gauge needle as previously described (20). Pre-surgical and post-surgical analgesics with buprenorphine (0.05 mg/kg, Sigma-Aldrich) and meloxicam (5 mg/kg, Sigma-Aldrich)were administered.

### Echocardiography

For transthoracic echocardiography, mice were anesthetized with 1% inhalational isoflurane and imaged using the Vevo 2100 High-Resolution Imaging System (Visual Sonics Inc.) with the MS-550 40 MHz probe. Measurements were obtained from M-mode sampling, and integrated EKV images were taken in the LV short axis at the mid-papillary level.

### Histological analysis

Short-axis heart sections from the mid ventricle were fixed in PBS/4% paraformaldehyde and embedded in paraffin. Fibrosis was visualized using Gomori’s Trichrome staining kit (Sigma-Aldrich) with quantification of the fibrotic area using ImagePro software. The cardiomyocyte cross-sectional area was determined by staining with WGA Alexa 488 (Invitrogen W11261) and analyzed using ImageJ (National Institutes of Health).

### Cell isolation from mice heart

Mouse cardiomyocytes isolation was performed by langendorff perfusion method, which was described in details previously (21). Cardiac fibroblast and endothelial cell isolation was performed using MACS cell separation (Miltenyi Biotech) following the manufacturer’s instructions. Briefly, the mouse heart was dissected and minced into small pieces. Collagenase type I (Worthington LS004196) was used for digestion in 37°C for 1 h. After 1 h incubation, a strainer was used to remove large particle and undigested tissues. RBC lysis buffer (ab204733) was used to remove red blood cells. Next, CD45 microbeads (Miltenyi Biotec, 130-052-301) and MS column (Miltenyi Biotec, 130-042-201) were used to remove leukocytes. To isolate cardiac fibroblast, CD90.2 microbeads (Miltenyi Biotec, 130-049-101) and MS were used. CD90.2 positive cells were collected from MACS column. CD31 microbeads (Miltenyi Biotec, 130-097-418) and MS column were used to isolate endothelial cells from flow through samples. The enrichment of the target cells was validated by qRT-PCR.

### Reverse transcription and quantitative real-time PCR

Total RNA was extracted using RNeasy Mini Kit (Qiagen 74106) according to the manufacturer’s protocol. The concentration was measured by a microplate reader (FLUOstar Omega, BMG LABTECH, Ortenberg, Germany). cDNA was synthesized using a reverse transcription supermix (iScript, BIO-RAD 1708841, CA, United States). Quantitative real-time PCR was performed on QuantStudio 5 Dx Real-Time PCR Systems (Applied Biosystems, Thermo Fisher Scientific, Inc.) with 2× qPCRBIO Probe Blue Mix Lo-ROX (PCR Biosystems Inc.) and TaqMan universal probes (Roche). All primers used in this manuscript are listed in Supplementary Table 1. *Ppib* was used as a reference for normalization. The relative mRNA expression was calculated by the ΔΔCt method.

### Statistical analysis

Data were shown as means ± *SEM*. Comparisons were analyzed by Student’s *t*-test, one-way or two-way analysis of variance (ANOVA). Multiple comparisons were taken into account when necessary. All statistical analysis was performed on IBM SPSS Statistics 22.0 (Armonk, NY, United States) or GraphPad Prism (San Diego, CA, United States). *P* < 0.05 was considered statistically significant.

## Results

### Double deletion of REV-ERBα/β in cardiomyocytes led to severe cardiac dysfunction and ventricular dilation after pressure overload

**FIGURE 1:**
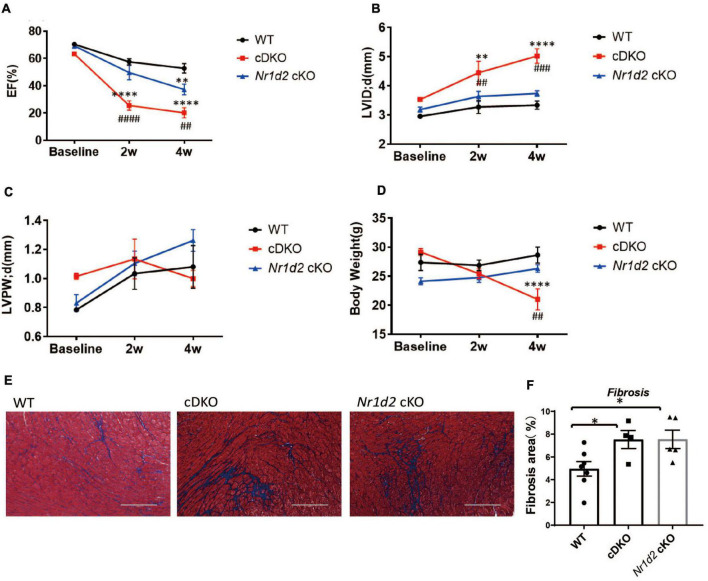
Deficiency of REV-ERBα/β or REV-ERBβ in cardiomyocytes exacerbates heart failure upon pressure overload. **(A–C)** Echocardiography analysis of ejection fraction (EF), LVID;d (left ventricle internal diameter; end-diastole), and LVPW;d (left ventricle posterior wall thickness; end-diastole) at __ weeks after TAC. **(D)** Body weight in WT, cDKO, and *Nr1d2* cKO mice. **(E,F)** Representative images and quantification of fibrosis area by Masson’s trichrome staining at 4 weeks after TAC. WT *n* = 10, cDKO *n* = 6, *Nr1d2* cKO *n* = 5. Data are shown as mean ± S.E.M. ^##^*p* < 0.01, ^###^*P* < 0.001, ^####^*P* < 0.0001, **p < 0.01, ***P < 0.0001 by two-way ANOVA, *indicates comparison to WT, ^#^indicates comparison to cDKO. Tukey’s test was used for multiple comparison corrections.

We generated cardiomyocyte-specific REV-ERBα/β double knockout mice, referred to as cDKO, by crossbreeding *Nr1d1/2^fl/fl^* mice (*Nr1d1*^TM 1.2Rev^, MGI ID 5426700 and *Nr1d2*^TM 1.1Rev^, MGI ID 5426699) with the αMHC-Cre line (22). Cre negative *Nr1d1/2^fl/fl^* littermates were used as WT controls. cDKO mice develop age-dependent dilated cardiomyopathy, as we recently reported. However, the cardiac stress response and pathological remodeling after pressure overload have not been studied in these mice (13, 14). cDKO mice did not show significant ventricular dilation or contractile dysfunction before the age of 20 weeks. Therefore, we performed the transverse aortic constriction (TAC) from 9 to 13 weeks of age when the cardiac structure and function were indistinguishable to the controls (Supplementary Figures 1A–I). We show that the cDKO mice were highly sensitive to pressure overload and had a significant drop in ejection fraction (EF) as early as 2 weeks after the surgery, with an average EF of 20.9 vs. 53.5% in WT controls (Figure 1A); this is accompanied by a significant left ventricle dilation at 5.02 vs. 3.33 mm in the controls (Figure 1B). The left ventricular (LV) dimension (LVID;d) was also increased in cDKO mice compared to WT controls, with no change for LV wall thickness (Figure 1C). We had to terminate the experiment at 4 weeks after surgery, as the cDKO mice showed a significant drop in body weight (21.0 g in cDKO vs. 28.6 g in WT controls) and reached the humane endpoint (Figure 1D). Histology with trichrome staining showed significantly increased fibrosis in cDKO compared to the WT mice (Figures 1E,F). WGA staining analysis of the cross-section area of muscle fiber did not reveal obvious changes in cDKO vs. WT mice, which supports eccentric hypertrophy or dilated cardiomyopathy as opposed to concentric hypertrophy (Supplementary Figures 1N,O). Thus, cardiac REV-ERB is essential for cardiac stress response and remodeling upon pressure overload since cDKO mice are highly susceptible to dilated cardiomyopathy in response to pressure overload.

### Single deletion of REV-ERBβ in cardiomyocytes showed mild cardiac dysfunction after pressure overload

To distinguish the functional significance between REV-ERBα and REV-ERBβ, we used a similar strategy to generate REV-ERBβ single cardiac deletion (*Nr1d2^f/f^*: α-*MHC-cre*), referred to as *Nr1d2* cKO. In contrast to cDKO, *Nr1d2* cKO showed a modest reduction in EF (36.2% in cKO vs. 20.9% in cDKO) at 4 week after TAC (Figure 1B). cKO mice did not show significant chamber dilation or LV wall thinning compared to WT mice (Figures 1C,D). Consistent with echocardiography analysis, *Nr1d2* cKO mice were able to maintain their body weight and normal activities on physical exams during the entire experiment (Figure 1A). However, trichrome staining showed significantly increased fibrosis in cKO heart compared to WT (Figures 1E,F), indicating that REV-ERBβ has an indispensable role on its own during pressure overload. The mild phenotypic changes in *Nr1d2* cKO mice suggest that REV-ERBα and REV-ERBβ could have largely redundant roles or REV-ERBα is the dominant isoform in the heart.

### SR9009 alleviates pressure overload-induced heart failure in the absence of cardiac REV-ERBs

**FIGURE 2:**
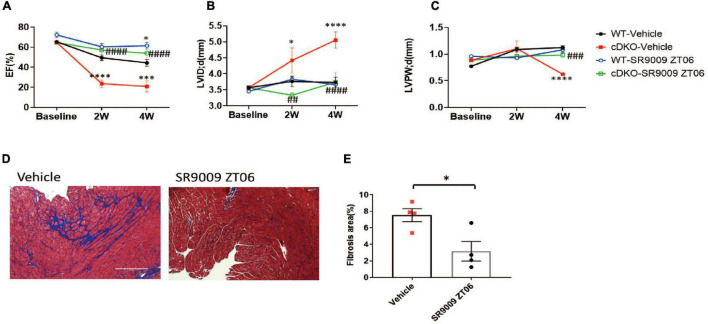
SR9009 rescues post-TAC cardiac dysfunction in REV-ERB deficient mice. **(A–C)** Echocardiography of WT and cDKO after vehicle or SR9009 treatment at ZT06. WT-vehicle *n* = 10, cDKO-vehicle *n* = 5, WT-SR9009 *n* = 5, and cDKO-SR9009 *n* = 6. **(D,E)** Representative images and quantification of fibrosis area by Masson’s trichrome staining at 4 weeks after TAC for cDKO mice treated with SR9009 at ZT06. Data are shown as mean ± S.E.M. ^##^*p* < 0.01, ^###^*P* < 0.001, ^####^*P* < 0.0001, *p < 0.05, ***p < 0.001, ****P < 0.0001 by two-way ANOVA, *indicates comparison to WT-vehicle, ^#^indicates comparison to cDKO-vehicle. Tukey’s test was used for multiple comparison corrections.

SR9009 is a widely used REV-ERB agonist that targets both REV-ERBα and REV-ERBβ. We have shown that SR9009 is cardioprotective in WT mice after pressure overload when administered at Zeitgeber time 6 (ZT6), a time point immediately before REV-ERB peak expression in the heart (10). To test if the SR9009 effects are through REV-ERB, we administered SR9009 to cDKO mice one day after TAC at ZT6. SR9009 still protected the cDKO mice from cardiac dysfunction, just as in the WT mice (Figures 2A–C and Supplementary Figures 2A–D). EF was normalized from 20.9% in the vehicle-treated group to 53.8% in the SR9009 ZT6 group at 4 weeks after TAC (Figure 2A). SR9009 also prevented the dilation of the left ventricle in cDKO hearts (LVID; d 5.06 mm with SR9009 vs. 3.74 mm with a vehicle in cDKO) (Figure 2B). The LV wall thickness also improved (LVPW;d 0.98 mm with SR9009 vs. 0.62 mm with a vehicle in cDKO) (Figure 2C). Trichrome staining revealed a reduction in cardiac fibrosis after SR9009 in cDKO mice (Figures 2D,E). Thus, SR9009 retains the full cardioprotective capacity in cDKO mice after TAC, demonstrating that the cardioprotective effect of SR9009 against pressure overload is not dependent on REV-ERB in cardiomyocytes.

### SR9009 alleviates pressure overload-induced heart failure similarly at different times of the day

**FIGURE 3:**
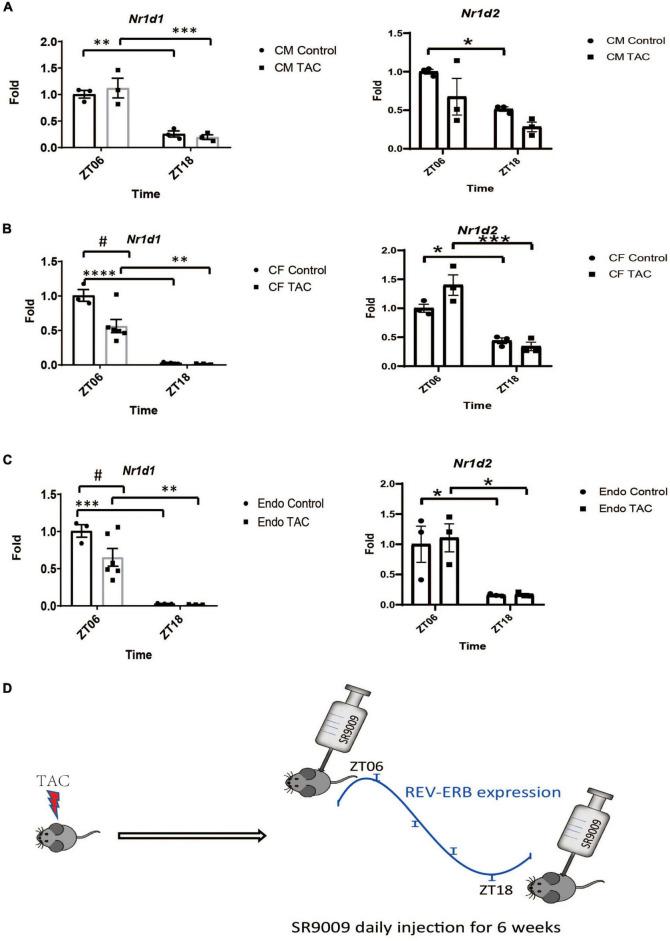
The rhythmic expression of REV-ERB in the major cell types of the heart. **(A–C)** Relative mRNA expression levels of *Nr1d1* and *Nr1d2* in major cell types in the heart (CM-cardiomyocytes, CF-cardiac fibroblasts and Endo-endothelial cells) isolated from mouse hearts at 6 weeks after TAC or sham surgery at ZT6 or ZT18. ^#^*p* < 0.05, *P < 0.05, **p < 0.01, ***P < 0.001 by two-way ANOVA. Tukey’s test was used for multiple comparison corrections. *n* = 3. **(D)** Diagram of the SR9009 treatment schemes. Mice receive daily SR9009 injection post-TAC at ZT06 or ZT18 for 6 weeks. ****P < 0.0001.

As a core component of the circadian clock, REV-ERBα expression in the heart oscillates robustly, this was validated by our RNA sequencing result in the mouse heart (14) as well as an independent previously published microarray study (GSE36407) (Supplementary Figures 3A–D). REV-ERBβ has a similar phase to REV-ERBα with a smaller amplitude of oscillation (Supplementary Figures 3A–D). We examined the expression of REV-ERBα and β in each of the main cell types in the adult mouse heart, including cardiomyocytes, cardiac fibroblasts, and cardiac endothelial cells at baseline and TAC conditions. The robust oscillatory pattern of REV-ERB was retained in all cell types tested, with ZT6 close to the expression peak and ZT18 close to the expression trough (Figure 3). While TAC does not change REV-ERB expression in the heart globally, when we examined each individual cell types, REV-ERBα expression is reduced by about 50% in cardiac fibroblasts and endothelial cells but not the cardiomyocytes (Figures 3A–C and Supplementary Figures 3A,B). So, the bulk RNA expression in the heart primarily reflects gene expression in the cardiomyocytes. At ZT18, REV-ERBα expression is at 1–2% compared to ZT6 for cardiac fibroblasts or cardiac endothelial cells and about 20% compared to ZT6 for cardiomyocytes (Figures 3A–C), which is comparable to what can be achieved by Cre deletion or siRNA knockdown. REV-ERBβ also shows a significant reduction pattern in the baseline condition of all cell types detected at ZT18 (Figures 3A–C). Thus, ZT18 is a time point when REV-ERB expression is sufficiently low in all major cardiac cell types that it creates a transient *de facto* KO or knockdown condition in the entire heart. Given the short half-life of SR9009 (2 h), we hypothesized that if SR9009 has a major effect on other non-myocyte cell types in the heart, its effects may be diminished when administered at a different time point (ZT18). In fact, a previous report showed the cardioprotective effect of SR9009 upon myocardial infarction was only evident at ZT6 and not evident at ZT18, which supports this notion (11).

**FIGURE 4:**
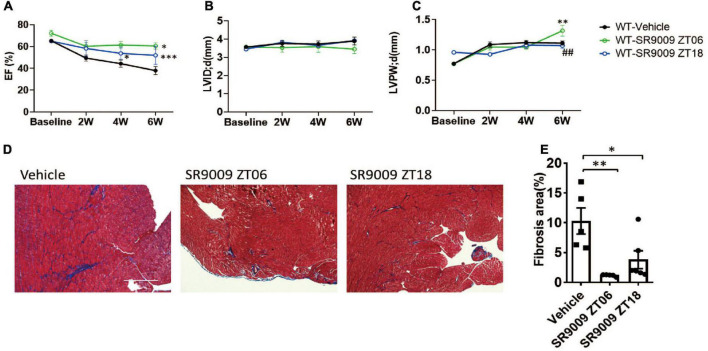
Post-TAC SR9009 treatment at ZT6 or ZT18 is equally cardioprotective. **(A–C)** Echocardiography analysis of time-dependent effect of SR9009 on cardiac functions in WT TAC mice. WT-vehicle *n* = 10, WT-SR9009 ZT06 *n* = 6, WT-SR9009 ZT18 *n* = 5. Data are shown as mean ± S.E.M. ^##^*p* < 0.01 by two-way ANOVA comparing to the WT-SR9009 ZT18 group, *P < 0.05, **p < 0.01, ***P < 0.001 by two-way ANOVA comparing to the WT vehicle group. Tukey’s test was used for multiple comparison corrections. **(D,E)** Representative images and quantification of fibrosis area by Masson’s trichrome staining at 6 weeks after TAC. *P < 0.05, **p < 0.01 by one-way ANOVA. Tukey’s test was used for multiple comparison corrections.

We then performed TAC surgery in WT mice and treated them with daily SR9009 at ZT18 (Figure 3D), when REV-ERB expression is very low in all cardiac cell types. Surprisingly, the effects of SR9009 at ZT18 were comparable to those at ZT6 as we had previously published (10). SR9009 given at ZT18 rescued TAC-induced cardiac dysfunction to a similar degree as SR9009 given at ZT6 (Figure 4A). Both showed EF in the normal range after 6 weeks of TAC (52.0 and 60.6% individually), significantly higher than the vehicle-treated group at 38% (Figure 4A). SR9009 did not seem to alter the chamber size or wall thickness of left ventricle (Figures 4B,C and Supplementary Figures 4A–D), but ameliorated fibrosis drastically (Figures 4D,E). The similar cardioprotective effects of SR9009 administrated at the peak and trough of REV-ERB expression suggest a REV-ERB-independent mechanism of SR9009 in counteracting pressure overload-mediated contractile dysfunction.

## Discussion

The cardioprotective function of REV-ERB was first established using the pharmacological tool drug SR9009 (10, 11). Although SR9009 can have non-specific targets (15), the essential role of REV-ERB in the heart was confirmed by more recent reports using two independent REV-ERB cardiac-specific knockout murine models (13, 14). To specifically investigate the role of REV-ERB in cardiac disease remodeling processes, we challenged REV-ERB cardiac-specific knockout mice (cDKO) with pressure overload. Our results from the genetic model demonstrated the key protective role of REV-ERB in cardiac pressure overload in addition to maintaining normal physiological homeostasis.

*Nr1d1* and *Nr1d2* genes that encode REV-ERBα and REV-ERBβ, respectively, share a high degree of homology (16, 17). *Nr1d1* has been demonstrated to be the dominant isoform in most systems studied to date, with partially redundant functions between the two (17–19). To investigate the contribution of REV-ERBα and REV-ERBβ in the heart, we studied the *Nr1d2* single cardiac cKO and compared it to cDKO. We found that *Nr1d2* cKO mice only show mild dysfunction when compared to the cDKO mice under TAC stress, indicating that *Nr1d2* alone is dispensable for cardiac protection upon pressure overload. Therefore, *Nr1d1* is the dominant isoform in the heart, or the two isoforms have largely redundant roles.

Considering the potential off-target effects of SR9009, we set out to evaluate its target specificity in the heart. We used the same regime we previously treated WT mice with, and found, SR9009 retains the full cardioprotective capacity in cDKO mice. The *Nr1d1* deletion allele results in an in-frame deletion of the DNA binding domain and a mutant protein, which may be able to “tether” to other transcription factors for target recognition (9). The *Nr1d2* deletion allele is a complete loss of function frameshift allele where no protein is expressed. One could argue that the truncated REV-ERBα might mediate the SR9009 effects. However, another cardiac REV-ERBα/β double KO mouse line with a frameshift *Nr1d1* deletion and a complete loss-of-function allele has an almost identical phenotype to the cDKO mice used in this study (13, 14), suggesting that the truncated REV-ERBα unlikely has a cardiac function.

As SR9009 is systemically administered, the effect of SR9009 may depend on REV-ERB in other cell types, as cardiomyocytes account for 30–50% of the number of cells in a healthy heart (23) and probably even lower proportion in a heart with fibrosis or inflammation. By carefully isolating various major cell types in the heart, we found that all cell types tested have the same phase for the oscillatory REV-ERB expression. This allowed us to administer SR9009 at a time when REV-ERB expression nadirs in all cell types. As our previous experiments were designed to administer SR9009 to match the peak of REV-ERB and capture the maximum target availability, we chose to also administer SR9009 at ZT18 when minimum REV-ERB is expressed. We found that SR9009 is equally effective at ZT6 or ZT18 despite the huge difference of REV-ERB expression levels between these two time-points in all major cell types in the heart. Therefore, SR9009 can have cardioprotective effects independent of REV-ERB. We realized that we have only examined 2 time points (the peak and the nadir of REV-ERB), additional time points in a 24-h day and increased number of animals may allow detection of more subtle differences in the timing of SR9009 treatment. Given its robust cardioprotective effects in multiple disease models, it will be interesting to explore the genuine targets of SR9009 in the future.

In conclusion, we demonstrate the cardioprotective role of the core circadian clock component REV-ERB in the pressure overload disease model. REV-ERVβ is largely dispensable in this process. SR9009 likely protects the heart through REV-ERB independent mechanisms, which warrants further investigations.

## Data availability statement

Publicly available datasets were analyzed in this study. This data can be found here: https://www.ncbi.nlm.nih.gov/geo/, GSE152372 and GSE36407.

## Ethics statement

The animal study was reviewed and approved by the Institutional Animal Care and Use Committee at Baylor College of Medicine.

## Author contributions

LZ, ZS, and TB conceived the project. HL, SS, C-lT, LQ, AG, EN, and YZ performed the experiment and analyzed the data. HL, YZ, ZS, and LZ wrote the manuscript with contributions from others. All authors contributed to the article and approved the submitted version.

## Conflict of interest

The authors declare that the research was conducted in the absence of any commercial or financial relationships that could be construed as a potential conflict of interest.

## Publisher’s note

All claims expressed in this article are solely those of the authors and do not necessarily represent those of their affiliated organizations, or those of the publisher, the editors and the reviewers. Any product that may be evaluated in this article, or claim that may be made by its manufacturer, is not guaranteed or endorsed by the publisher.

## Footnotes

1
http://www.informatics.jax.org/allele/MGI:5426699

## Supplementary material

Supplementary Figure 1 Echocardiography parameters and histology analysis in WT, cDKO, and Nr1d2 cKO mice in Sham and TAC groups. **(A–H)** Echocardiography analysis of EF, FS (fractional shortening), LVID;s (left ventricular internal dimension end-systole), LVID;d (left ventricle internal diameter; end-diastole), LVPW;s (left ventricle posterior wall thickness; end systole), LVPW;d (left ventricle posterior wall thickness; end-diastole), LV Mass AW (corrected) (corrected left ventricular mass) and Body weight in WT. **(I)** Representative images and quantification of fibrosis area by Masson’s trichrome staining of Sham animal hearts at 13 weeks. WT *n* = 3, cDKO *n* = 3. **(J–M)** Echocardiography analysis of FS (fractional shortening), LVID;s (left ventricular internal dimension end-systole), LV Mass AW (corrected) (corrected left ventricular mass), and LVPW;s (left ventricle posterior wall thickness; end systole) in WT, cDKO and *Nr1d2* cKO mice after TAC. WT *n* = 10, cDKO *n* = 6, *Nr1d2* cKO *n* = 5. Data are shown as mean ± S.E.M. ^#^*p* < 0.05, ^##^*p* < 0.01, ^####^*P* < 0.0001, ***p* < 0.01, *****P* < 0.0001 by two-way ANOVA, *indicates comparison to WT, ^#^indicates comparison to cDKO. Tukey’s test was used for multiple comparison corrections. **(N,O)** Representative images and quantification of cross-section area by WGA (Wheat Germ Agglutinin) staining *n* = 4.

Supplementary Figure 2 Echocardiography analysis of post-TAC cardiac protective effect of SR9009 in WT TAC and cDKO mice. **(A–D)** Echocardiography analysis of cardiac protective effect of SR9009 in WT and cDKO mice before and after TAC. FS, LVID;s, LV Mass AW (corrected) and LVPW;s were shown. WT-vehicle *n* = 10, cDKO-vehicle *n* = 5, WT-SR9009 ZT06 *n* = 5, and cDKO-SR9009 ZT06 *n* = 6. Data are shown as mean ± S.E.M. ****p* < 0.001, *****P* < 0.0001, ^##^*p* < 0.01, ^###^*P* < 0.001, ^####^*P* < 0.0001, ***p* < 0.01, *** *p* < 0.001, *****P* < 0.0001 by two-way ANOVA, *indicates comparison to WT-vehicle, # indicates comparison to cDKO-vehicle. Tukey’s test was used for multiple comparison corrections.

Supplementary Figure 3 *Nr1d1* expression from GSE36407. **(A,B)** Relative mRNA expression levels of *Nr1d1* and *Nr1d2* in mouse hearts at multiple Zeitgeber times (ZTs) in Sham (Curve shows in Black) and TAC (Curve shows in Red) conditions. Raw data is from GSE36407 in GEO (Gene Expression Omnibus) **P* < 0.05, ****P* < 0.001, by two-way ANOVA, *indicates comparison to sham group. **(C,D)** Relative mRNA expression levels of *Nr1d1* and *Nr1d2* in mouse hearts at multiple Zeitgeber times (ZTs) with data from previous RNAseq study (14). **(E)** Relative mRNA expression levels of cardiomyocytes (*Myh6*), cardiac fibroblasts (*Col1a1*), and cardiac endothelial (*CDH5*) marker genes in the isolated cells from mice hearts. **P* < 0.05, ***p* < 0.01, ****P* < 0.001, *****P* < 0.0001, by one-way ANOVA, *indicates comparison between cell types.

Supplementary Figure 4 Echocardiography analysis of the role of SR9009 on post-TAC cardiac protection when administered at different time points. **(A–D)** Echocardiography analysis. FS, LVID;s, LV Mass AW (corrected), and LVPW;s after TAC were shown. WT-vehicle *n* = 10, WT-SR9009 ZT06 *n* = 6, WT-SR9009 ZT18 *n* = 5. Data are mean ± S.E.M. **P* < 0.05, ***p* < 0.01, ****P* < 0.001 by two-way ANOVA, *indicates comparison to WT vehicle group. Tukey’s test was used for multiple comparison corrections.
