# Supplementary figures and images for: SR9009 improves heart function after pressure overload independent of cardiac REV-ERB
Source: Front Cardiovasc Med. 2022 Jul 14;9:952114. doi: 10.3389/fcvm.2022.952114 (PMC9329699; doi:10.3389/fcvm.2022.952114)

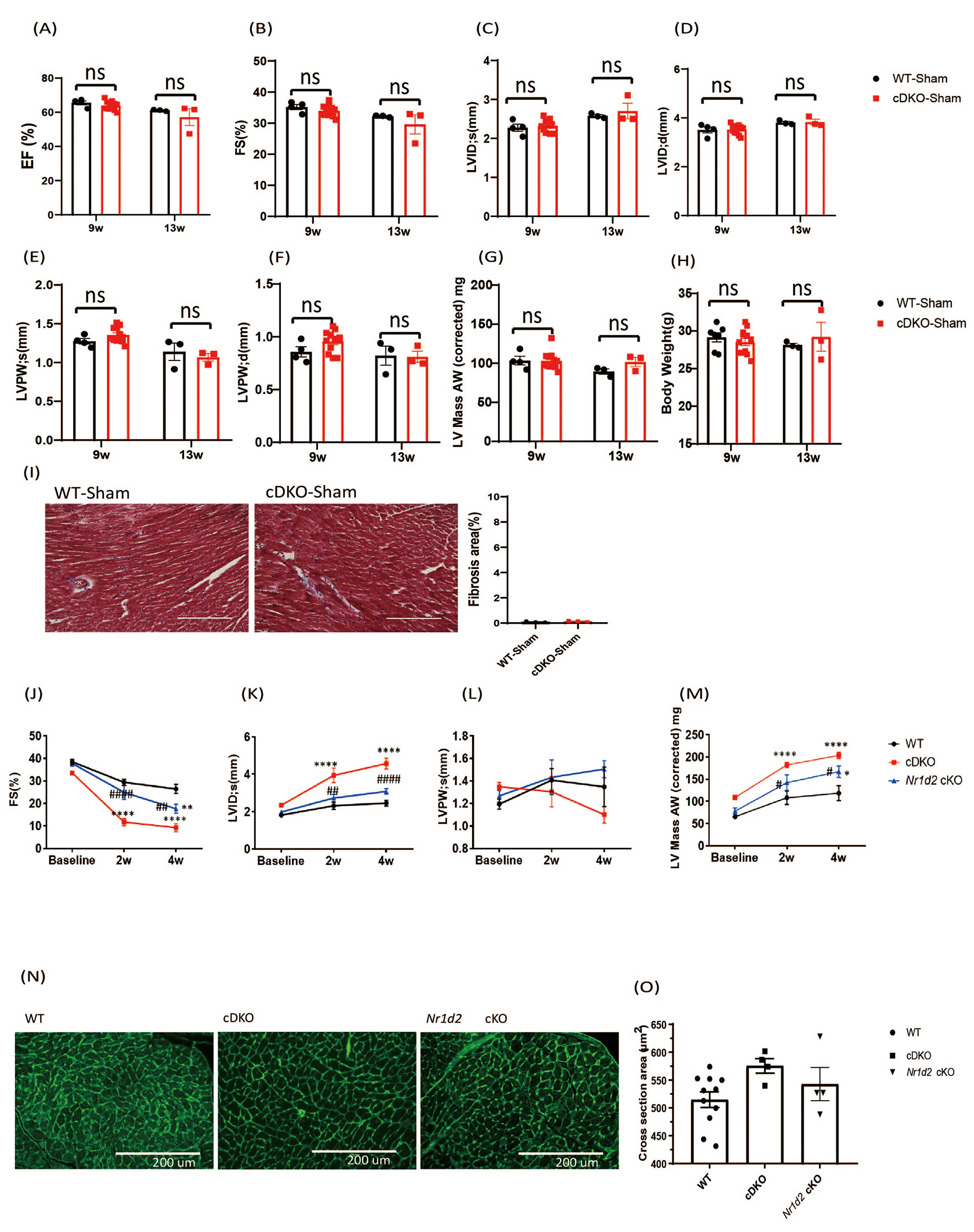

Supplement: Supplementary Figure 1 — Echocardiography parameters and histology analysis in WT, cDKO, and Nr1d2 cKO mice in Sham and TAC groups. (A–H) Echocardiography analysis of EF, FS (fractional shortening), LVID;s (left ventricular internal dimension end-systole), LVID;d (left ventricle internal diameter; end-diastole), LVPW;s (left ventricle posterior wall thickness; end systole), LVPW;d (left ventricle posterior wall thickness; end-diastole), LV Mass AW (corrected) (corrected left ventricular mass) and Body weight in WT. (I) Representative images and quantification of fibrosis area by Masson’s trichrome staining of Sham animal hearts at 13 weeks. WT n = 3, cDKO n = 3. (J–M) Echocardiography analysis of FS (fractional shortening), LVID;s (left ventricular internal dimension end-systole), LV Mass AW (corrected) (corrected left ventricular mass), and LVPW;s (left ventricle posterior wall thickness; end systole) in WT, cDKO and Nr1d2 cKO mice after TAC. WT n = 10, cDKO n = 6, Nr1d2 cKO n = 5. Data are shown as mean ± S.E.M. #p < 0.05, ##p < 0.01, ####P < 0.0001, **p < 0.01, ****P < 0.0001 by two-way ANOVA, *indicates comparison to WT, #indicates comparison to cDKO. Tukey’s test was used for multiple comparison corrections. (N,O) Representative images and quantification of cross-section area by WGA (Wheat Germ Agglutinin) staining n = 4. [file Image_1.JPEG]

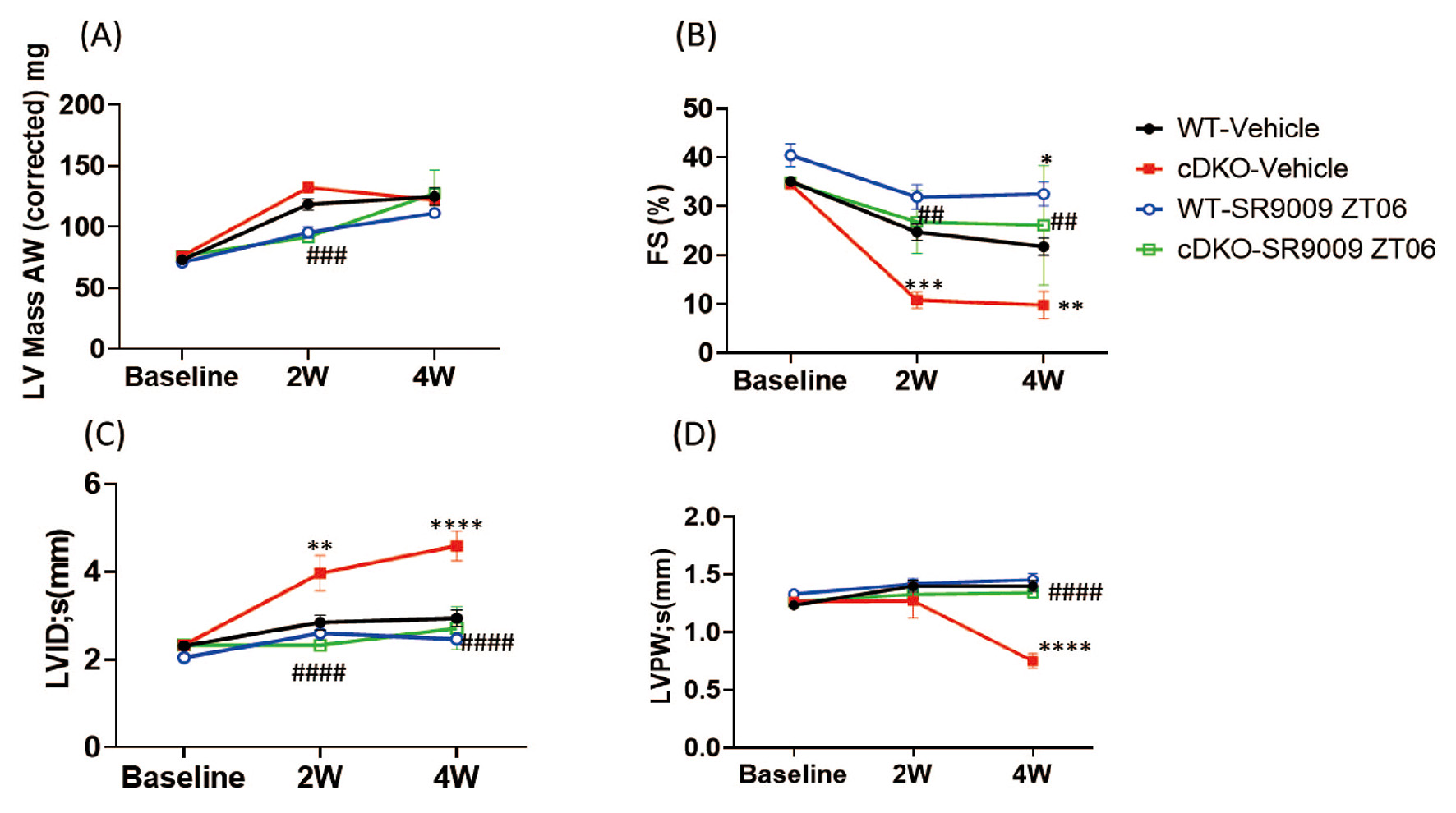

Supplement: Supplementary Figure 2 — Echocardiography analysis of post-TAC cardiac protective effect of SR9009 in WT TAC and cDKO mice. (A–D) Echocardiography analysis of cardiac protective effect of SR9009 in WT and cDKO mice before and after TAC. FS, LVID;s, LV Mass AW (corrected) and LVPW;s were shown. WT-vehicle n = 10, cDKO-vehicle n = 5, WT-SR9009 ZT06 n = 5, and cDKO-SR9009 ZT06 n = 6. Data are shown as mean ± S.E.M. ***p < 0.001, ****P < 0.0001, ##p < 0.01, ###P < 0.001, ####P < 0.0001, **p < 0.01, *** p < 0.001, ****P < 0.0001 by two-way ANOVA, *indicates comparison to WT-vehicle, # indicates comparison to cDKO-vehicle. Tukey’s test was used for multiple comparison corrections. [file Image_2.JPEG]

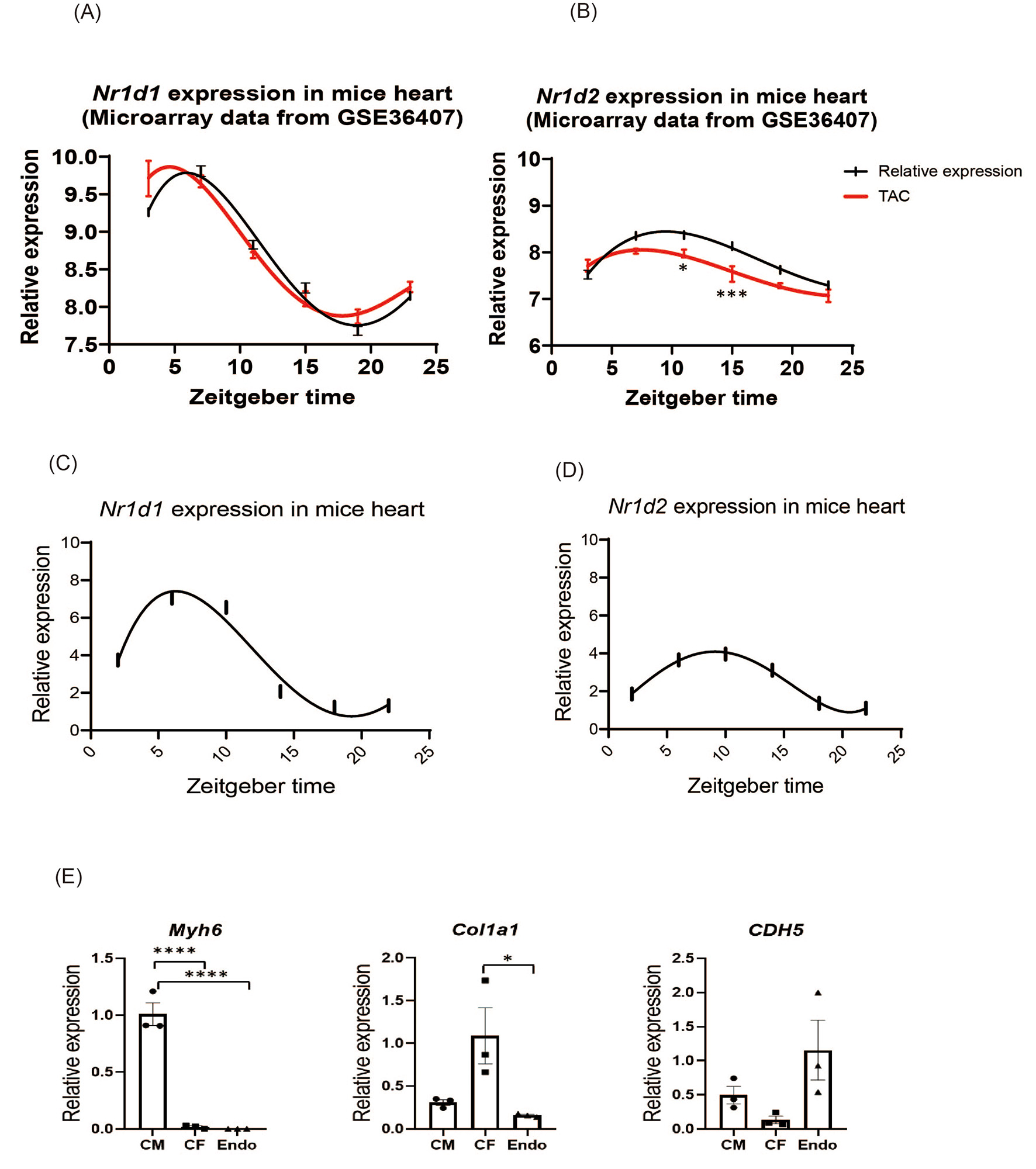

Supplement: Supplementary Figure 3 — Nr1d1 expression from GSE36407. (A,B) Relative mRNA expression levels of Nr1d1 and Nr1d2 in mouse hearts at multiple Zeitgeber times (ZTs) in Sham (Curve shows in Black) and TAC (Curve shows in Red) conditions. Raw data is from GSE36407 in GEO (Gene Expression Omnibus) *P < 0.05, ***P < 0.001, by two-way ANOVA, *indicates comparison to sham group. (C,D) Relative mRNA expression levels of Nr1d1 and Nr1d2 in mouse hearts at multiple Zeitgeber times (ZTs) with data from previous RNAseq study (14). (E) Relative mRNA expression levels of cardiomyocytes (Myh6), cardiac fibroblasts (Col1a1), and cardiac endothelial (CDH5) marker genes in the isolated cells from mice hearts. *P < 0.05, **p < 0.01, ***P < 0.001, ****P < 0.0001, by one-way ANOVA, *indicates comparison between cell types. [file Image_3.JPEG]

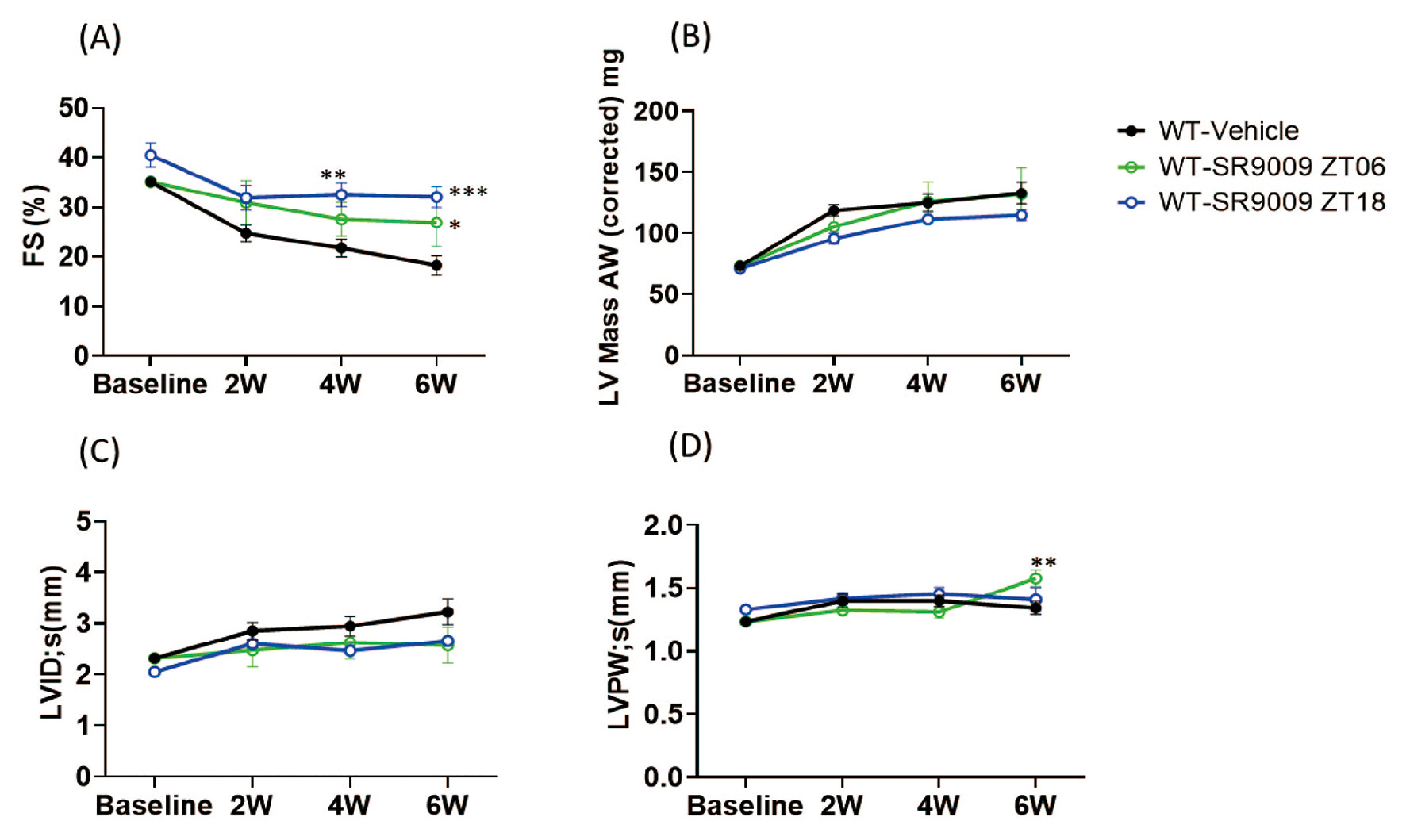

Supplement: Supplementary Figure 4 — Echocardiography analysis of the role of SR9009 on post-TAC cardiac protection when administered at different time points. (A–D) Echocardiography analysis. FS, LVID;s, LV Mass AW (corrected), and LVPW;s after TAC were shown. WT-vehicle n = 10, WT-SR9009 ZT06 n = 6, WT-SR9009 ZT18 n = 5. Data are mean ± S.E.M. *P < 0.05, **p < 0.01, ***P < 0.001 by two-way ANOVA, *indicates comparison to WT vehicle group. Tukey’s test was used for multiple comparison corrections. [file Image_4.JPEG]

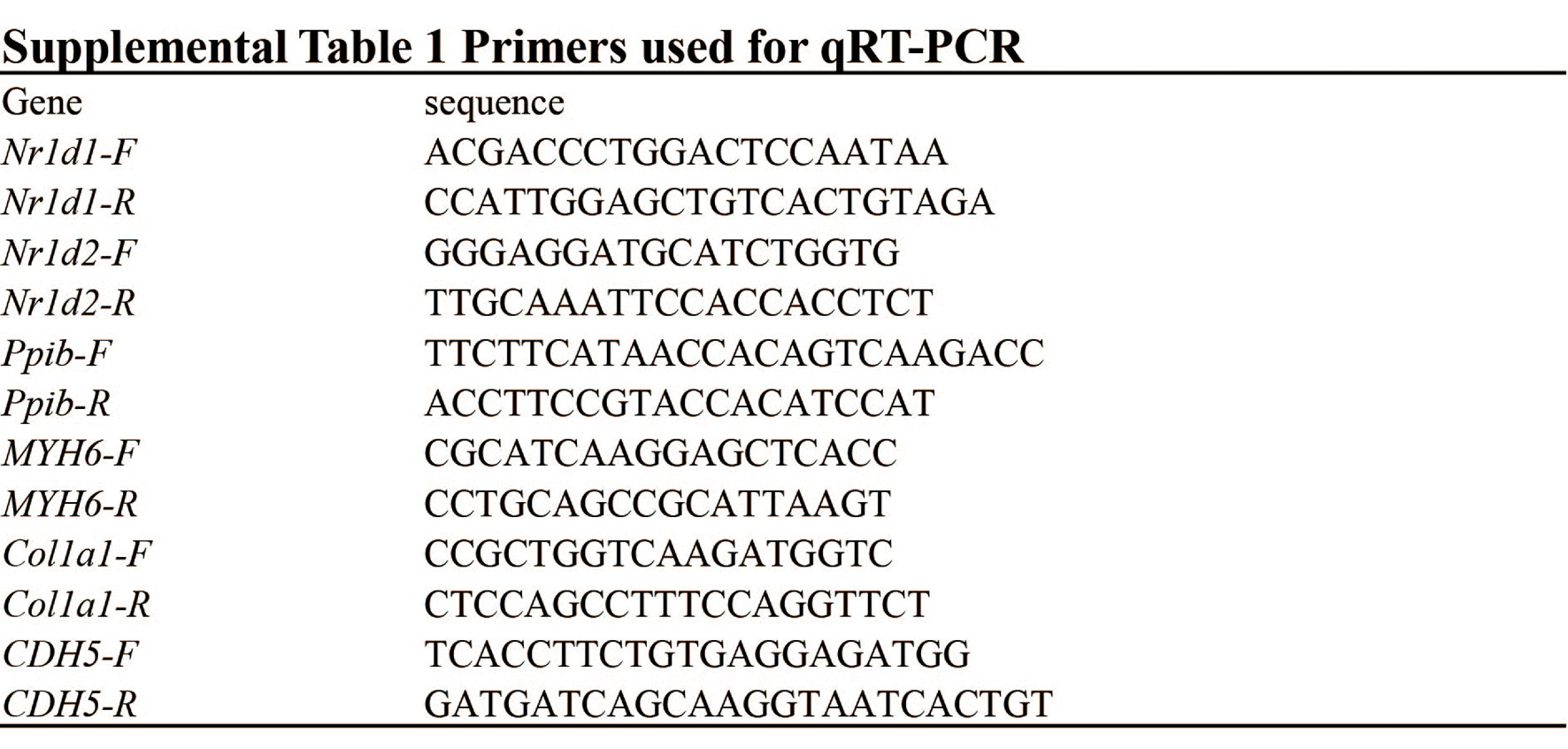

Supplement: Supplementary file 5 [file Image_5.JPEG]
